# A new and aberrant species of *Dugesia* (Platyhelminthes, Tricladida, Dugesiidae) from Madagascar

**DOI:** 10.3897/zookeys.425.7811

**Published:** 2014-07-15

**Authors:** Giacinta Angela Stocchino, Ronald Sluys, Renata Manconi

**Affiliations:** 1 Dipartimento di Scienze della Natura e del Territorio, Via Muroni 25, University of Sassari, I-07100, Italy; 2 Naturalis Biodiversity Center, P.O. Box 9517, 2300 RA Leiden, The Netherlands

**Keywords:** Tricladida, *Dugesia*, Madagascar, morphology, karyology, reproduction, new species

## Abstract

In this paper we report a new species of *Dugesia* of the family Dugesiidae from Madagascar, representing the fourth species of freshwater planarian known from this global biodiversity hotspot. In some respects the new species is aberrant, when compared with its congeners, being characterized by a head with smoothly rounded auricles, a peculiar course of the oviducts, including the presence of a common posterior extension, and by the asymmetrical openings of the vasa deferentia at about halfway along the seminal vesicle. Further, it is characterized by a ventral course of the ejaculatory duct with a terminal opening, very long spermiducal vesicles and unstalked cocoons. Its diploid chromosome complement with 18 chromosomes represents an uncommon feature among fissiparous species of *Dugesia*.

## Introduction

Madagascar, the fourth largest island in the world, is one of the priority global hotspots for biodiversity conservation (Mittermeier et al. 2004, Vieites et al. 2009) with 5% of the total species in the world and a very high percentage of endemism (70%). Unfortunately, this outstanding species richness is greatly endangered, due to rapidly increasing deforestation, soil erosion, and habitat destruction in large areas of the island (Harper et al. 2007, Andreone et al. 2008). This is particularly true for freshwater environments and their communities of benthic invertebrates.

It has been shown that Madagascar represents a global biodiversity hotspot for land planarians (Sluys 1998, 1999) but, unfortunately, freshwater triclads have received much less attention. With respect to freshwater planarians only representatives of the genus *Dugesia* Girard, 1850 of the family Dugesiidae Ball, 1974 have been reported from Madagascar. This genus is present with three species, two of which are endemic, viz. *Dugesia debeauchampi* De Vries, 1988, *Dugesia myopa* De Vries, 1988, and *Dugesia milloti* De Beauchamp, 1952 (cf. De Beauchamp 1952, De Vries 1988, Stocchino et al. 2002) (Table 1). The first species is known only from the type locality, Nosy Bé Island, north-west of Madagascar. *Dugesia myopa* is reported from two localities, viz. Andranoboka Cave, northwest of Mahajanga, and from Andringitra, in the southeast of the island. In contrast, *Dugesia milloti* is recorded from widely scattered Madagascan localities, viz. Montagne d’Ambre, Ivohibe, Morafenobé, and occurs also on the island of Anjouan in the Comoros Archipelago (De Beauchamp 1952, De Vries 1988).

**Table 1.** Annotated checklist of Madagascan freshwater triclads.

Taxa	Geographic Distribution and Habitat	References
Dugesiidae Ball, 1974		
*Dugesia* Girard, 1850		
*Dugesia bifida* Stocchino & Sluys, 2014	small unnamed tributary of the Mania River (type locality), southern branch of the High Tsiribihina hydrographic basin, western slope, south-east Madagascar, coll. R. Manconi, September 2011	present paper
*Dugesia debeauchampi* De Vries, 1988	Teyiamarango Stream (type locality), Nosy Bé Island, north Madagascar, coll. F. Starmühlner, 1958	De Vries 1988
*Dugesia milloti* De Beauchamp, 1952	Morafenobé, Mahajeby Forest (type locality), Manambaho hydrographic basin, western slope, central-west Madagascar, coll. M. Paulian; Ivohibe, eastern slope, south-east Madagascar, coll. J. Millot, 1950; Mutsamudu Fall, Anjouan, Comoros Archipelago, coll. J. Millot, October 1953; Roussette Stream, Montagne d’Ambre area, north-eastern slope and Ambre, north Madagascar, 17 September 1957	De Beauchamp 1952, De Vries 1988
*Dugesia myopa* De Vries, 1988	Zanadoa, Andringitra, south-east Madagascar, 1949; Andranoboka Cave (type locality), northwest of Mahajanga, Betsiboka hydrographic basin, western slope, north-west Madagascar, coll. M. Paulin, December 1951	De Beauchamp 1952, De Vries 1988

A recent field survey performed by one of us (R. Manconi) on the presence and distribution of Madagascan aquatic invertebrates in some unexplored lentic or lotic freshwaters of the High Plateau and the oriental slope (31 sites) yielded several new records of planarians, suggesting that species richness of this taxon in the island is underestimated.

In this paper we describe a new, and in some respects aberrant, species of *Dugesia* that was identified on the basis of morphological and karyological data. This contribution represents a first step of a more comprehensive faunistic and taxonomic study of planarian populations on Madagascar. An integrative taxonomic analysis, including molecular and morphological data, is in progress in order to analyze in more detail the problematic position of Madagascan freshwater triclads in an historical biogeographic scenario involving the splitting of Gondwana (cf. Sluys et al. 1998).

## Materials and methods

The collected specimens were transferred to the laboratory and were reared in glass bowls under semi-dark conditions at 18 +/- 2 °C; the worms were fed with fresh beef liver.

For morphological study specimens were fixed for 24 hours in Bouin’s fluid, dehydrated in an ascending ethanol series, cleared in clove oil, and embedded in synthetic paraffin. Serial sections were made at intervals of 6–8 µm and were stained with Mallory-Cason. Reconstructions of the copulatory apparatus were obtained by using a camera lucida attached to a compound microscope.

For karyological analyses metaphasic plates were obtained by the squashing method and also air drying (splashing), following Vacca et al. (1993). The squashing method was performed on single caudal regenerative blastemas of 10 specimens in order to verify the uniformity of the chromosome complement of the strain. The air drying method was performed on 15 intact specimens, thus yielding good metaphasic plates for karyometrical analysis. The chromosome complement was characterized on the basis of 6 metaphasic plates. Karyometric values were calculated after first arranging the chromosomes according to their gradually decreasing lengths. Relative length was calculated as chromosome length × 100/total length of the haploid genome. Centromeric index was calculated as length of short arm × 100/total length of the chromosome. Chromosomal nomenclature follows Levan et al. (1964).

The histological material is deposited at Naturalis Biodiversity Center, Leiden, The Netherlands (ZMA collection code), and in the Giacinta A. Stocchino collection (CGAS), University of Sassari.

### Abbreviations used in the figures

bc: bursal canal; ca: common atrium; cb: copulatory bursa; cg: cement glands; cm: circular muscle; cpe: common posterior oviducal extension; d: diaphragm; du: ductule; e: eye; ed: ejaculatory duct; epg: extra bulbar penial glands; g: gonopore; lm: longitudinal muscle; lob: left oviducal branch; lod: left oviduct; lvd: left vas deferens; ma: male atrium; o: ovary; od: oviduct; ov: oviducal vesicle; pb: penis bulb; pg: penial glands; ph: pharynx; pp: penis papilla; rod: right oviduct; rob: right oviducal branch; rvd: right vas deferens; s: sperm; sg: shell glands; sp: spermatophore; sv: seminal vesicle; spv: spermiducal vesicle; t: testes; tu: tuba; v: vitellarium.

## Results

### Systematic Account
Order Tricladida Lang, 1884
Suborder Continenticola Carranza, Littlewood, Clough, Ruiz-Trillo, Baguñà & Riutort, 1998
Family Dugesiidae Ball, 1974
Genus *Dugesia* Girard, 1850

#### Dugesia bifida

Taxon classificationAnimaliaTricladidaDugesiidae

Stocchino & Sluys sp. n.

http://zoobank.org/E72381C7-6FC3-422F-A8DA-1D4D0856B521

**Figure 1.**
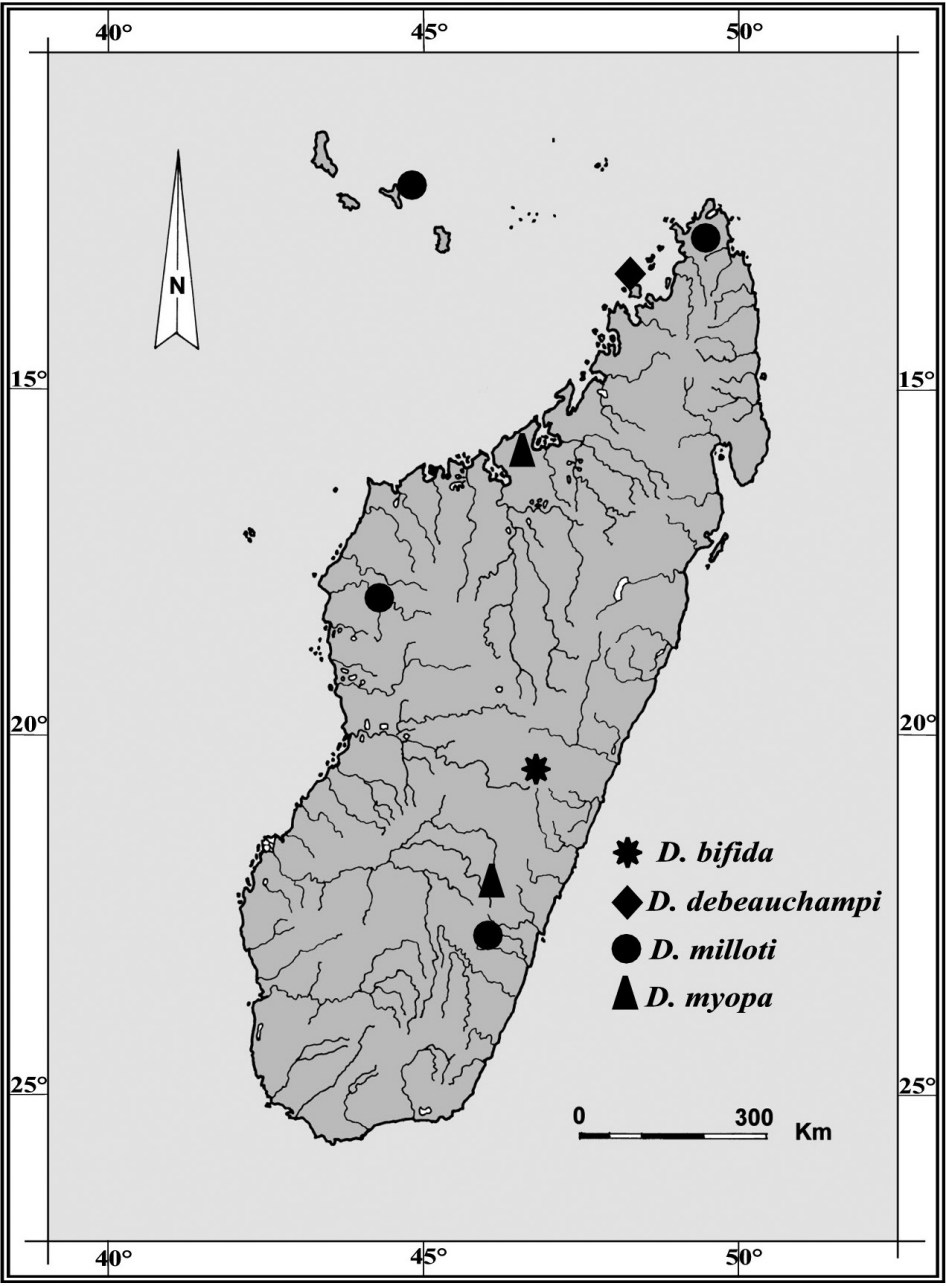
Geographic distribution of *Dugesia* species recorded from Madagascar and adjacent islands. Type locality of *Dugesia bifida* in the High Tsiribihina hydrographic basin indicated by an asterisk.

Figs 1 [Fig F2] [Fig F3] [Fig F4] [Fig F5] [Fig F6] –7 ; Tables 1 –2

##### Material examined.

Holotype: ZMA V.Pl. 7189.1, one set of sagittal sections on 8 slides, Central High Plateau, between Antsirabe (19°86'32"S, 47°03'36"E) and Ambositra (20°53'14"S, 47°24'61"E), near the small village of Antsariboti, Madagascar, 16 September 2011, coll. R. Manconi.

Paratypes: CGAS Pla 7.1, ibid., sagittal sections on 7 slides; CGAS Pla 7.2, ibid., sagittal sections on 4 slides; CGAS Pla 7.3, ibid., transverse sections on 20 slides. CGAS Pla 7.4-5, ibid., horizontal sections on 4, 7, slides respectively; ZMA V.Pl. 7189.2, ibid., horizontal sections on 6 slides; ZMA V.Pl. 7189.3, ibid., horizontal sections on 3 slides; ZMA V.Pl. 7189.4, ibid., horizontal sections on 4 slides; ZMA V.Pl. 7189.5, ibid., sagittal sections on 7 slides; ZMA V.Pl. 7189.6, ibid., horizontal sections on 5 slides.

##### Diagnosis.

*Dugesia bifida* is characterized by the presence of the following features: body slender; head with smooth, rounded auricles; oviducts that recurve before opening into the bursal canal and provided with a common posterior extension; slightly asymmetrical openings of the oviducts into the bursal canal; absence of ectal reinforcement; large seminal vesicle; asymmetrical openings of the vasa deferentia into the seminal vesicle, the openings situated at halfway along the vesicle; very long spermiducal vesicles; large diaphragm; ventral course of the ejaculatory duct; terminal opening of the ejaculatory duct; unstalked cocoons; chromosomal number 2n = 18.

##### Etymology.

The specific epithet is derived from the Latin adjective *bifidus*, split into two parts, and alludes to the fact that the peculiar long common oviduct splits into two branches, each branch subsequently opening into the bursal canal.

##### Geographical distribution.

Known only from the type locality in the High Tsiribihina hydrographic basin, Madagascar.

##### Habitat.

Planarians were found in running water in a paddy field area at an altitude ca. 1300 m asl in the Central High Plateau, along Route Nationale 7, between Antsirabe and Ambositra, near the small village of Antsariboti (Fig. 1). The small, unnamed stream is a tributary of the Mania River in the southern branch of the High Tsiribihina hydrographic basin. The animals, scattered and not abundant, were collected from running clear water, under small pebbles on coarse sand at a depth of 3–10 cm. A survey of ca. 50 pebbles, performed at the end of the dry season (September), revealed complete absence of planarian cocoons, as well as other invertebrates, excepting very small larvae of mayflies.

##### Description.

Body of living specimens slender, ranging from 6 to 7 mm in length and 0.4–0.6 mm in width in fissiparous specimens and from 11–15 mm × 1.5–2 mm in sexualized specimens. Two eyes present in the centre of the head; unpigmented auricular grooves marginally placed just posteriorly to the eyes. Head with smooth, rounded auricles and with five sensory fossae on either side of its anterior margin.

**Figure 2. F2:**
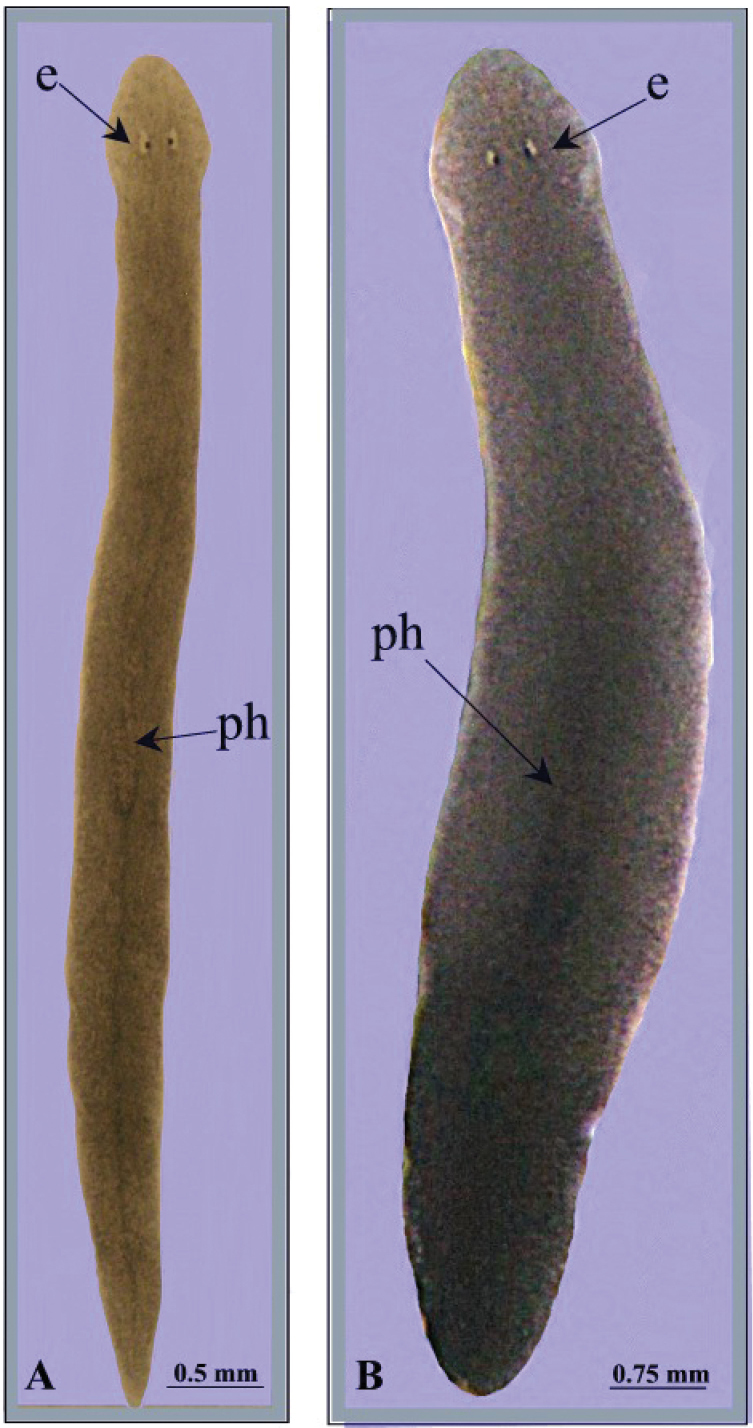
*Dugesia bifida*. Habitus of living specimens, **A** fissiparous individual **B** ex-fissiparous individual.

The dorsal surface light grey-brown, with two darker lateral stripes running from the central part of the pharynx to its posterior part, where they form a single median stripe that runs to the tail. In sexualized specimens the pigmentation is darker than fissiparous animals (Fig. 2). The ventral surface is paler than the dorsal body surface.

The pharynx is positioned in the posterior half of the body and measures about 1/9th of the body length. Inner and outer pharyngeal musculature bilayered, i.e. without an extra, third, outer longitudinal muscle layer in the inner sheath of muscles.

**Figure 3. F3:**
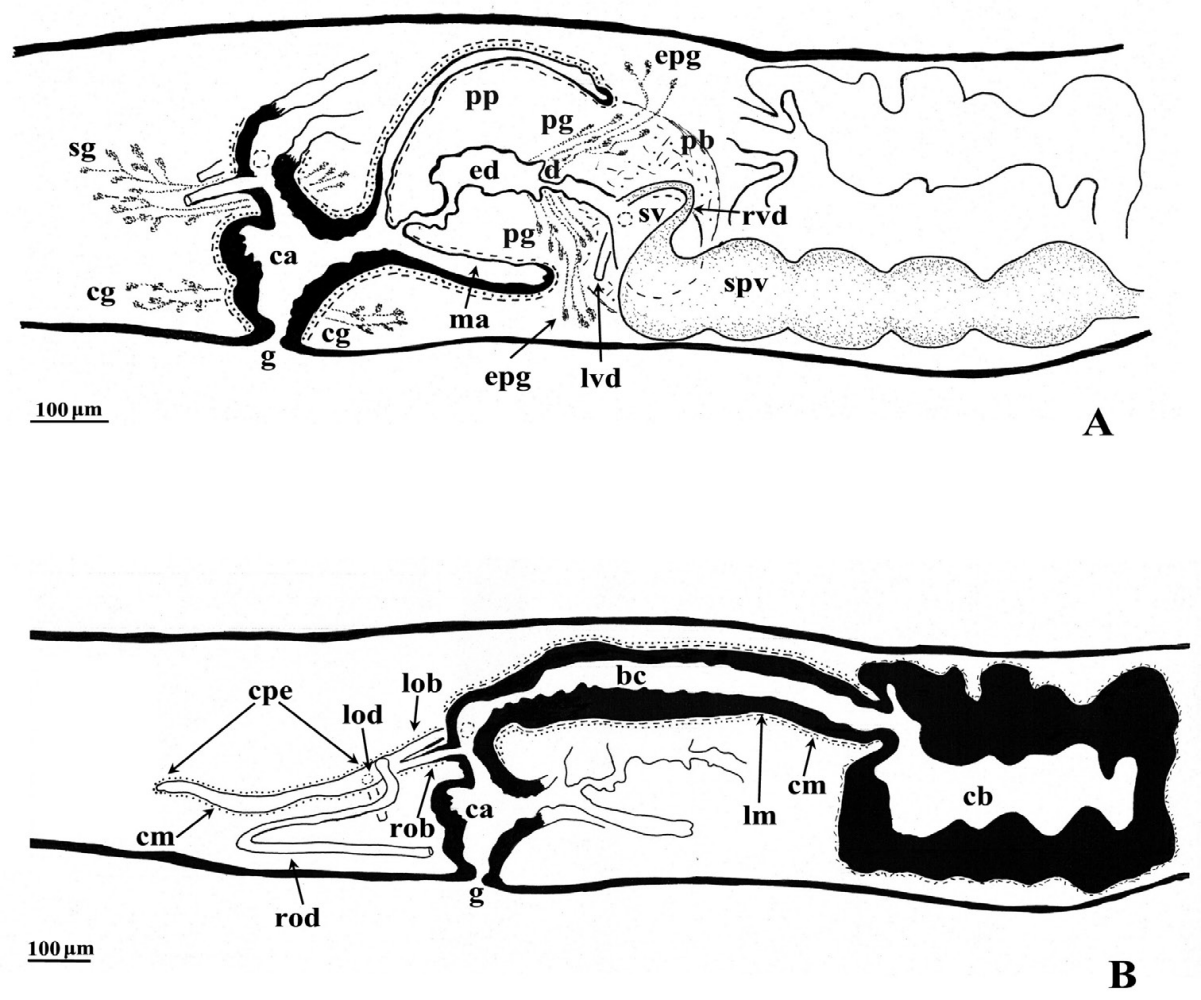
*Dugesia bifida*. Holotype ZMA V.Pl. 7189.1, sagittal reconstructions of the copulatory apparatus (anterior to the right), **A** male copulatory apparatus **B** female copulatory apparatus.

**Figure 4. F4:**
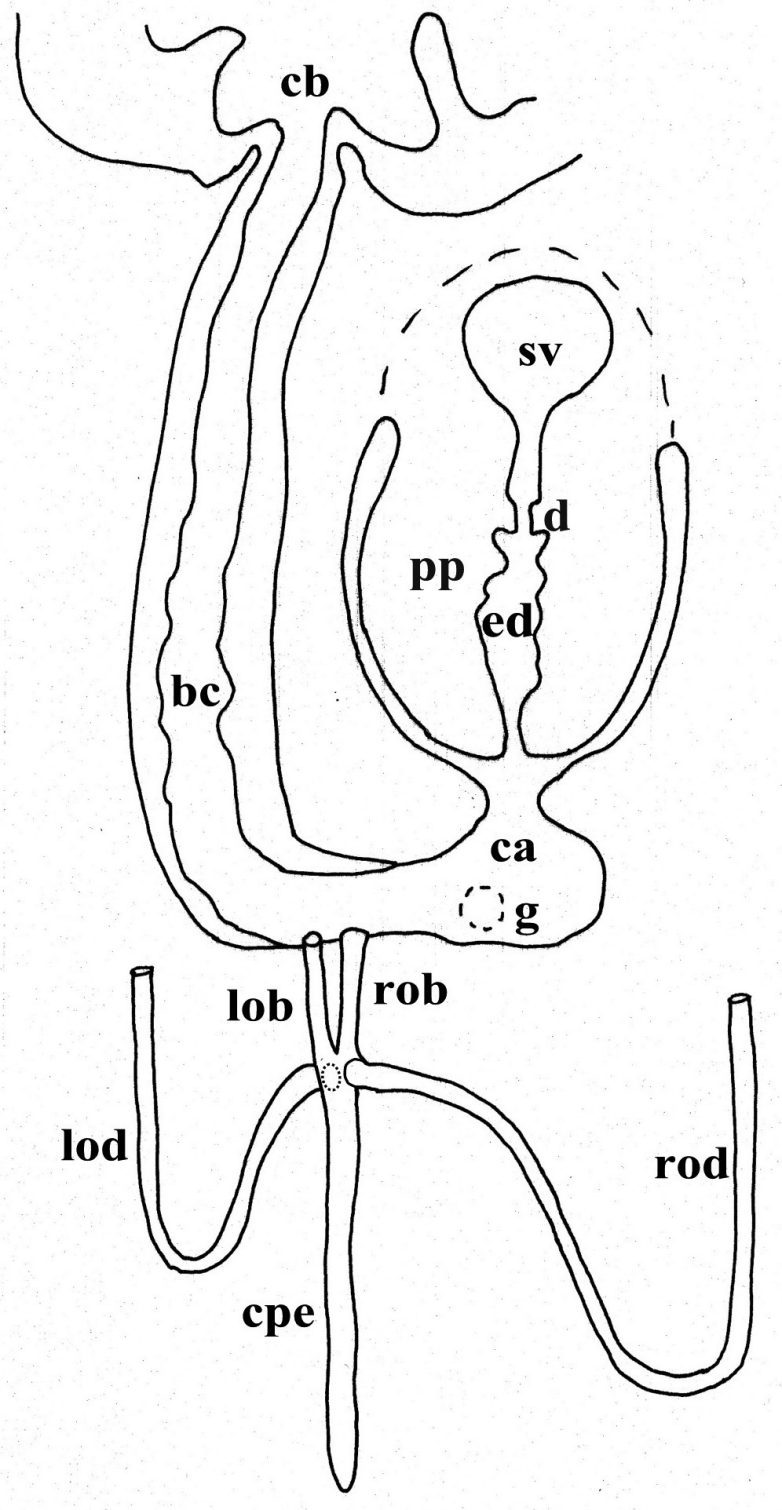
*Dugesia bifida*. Schematic horizontal reconstruction of the copulatory apparatus.

**Figure 5. F5:**
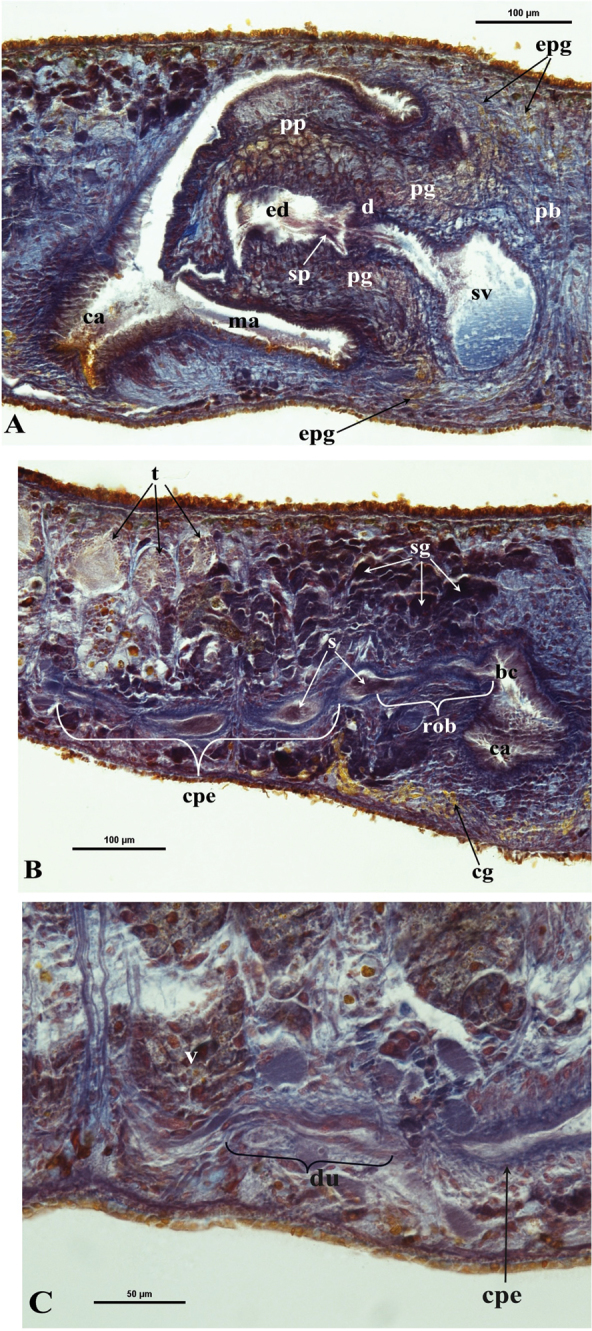
*Dugesia bifida*. Microphotographs of the copulatory apparatus. **A** Holotype ZMA V.Pl. 7189.1, sagittal section showing the penis bulb (pb) and the penis papilla (pp) with the seminal vesicle (sv) and the ejaculatory duct (ed) **B** Holotype ZMA V.Pl. 7189.1, sagittal section showing the opening of the right oviducal branch (rob) through the posterior wall of the bursal canal (bc), and the common posterior oviducal extension (cpe) full of sperm (s) **C** Paratype CGAS Pla 7.1, sagittal section showing the caudal part of the common posterior oviducal extension (cpe) and the ductule (du) communicating with the ventral part of an adjacent vitellarium (v).

**Figure 6. F6:**
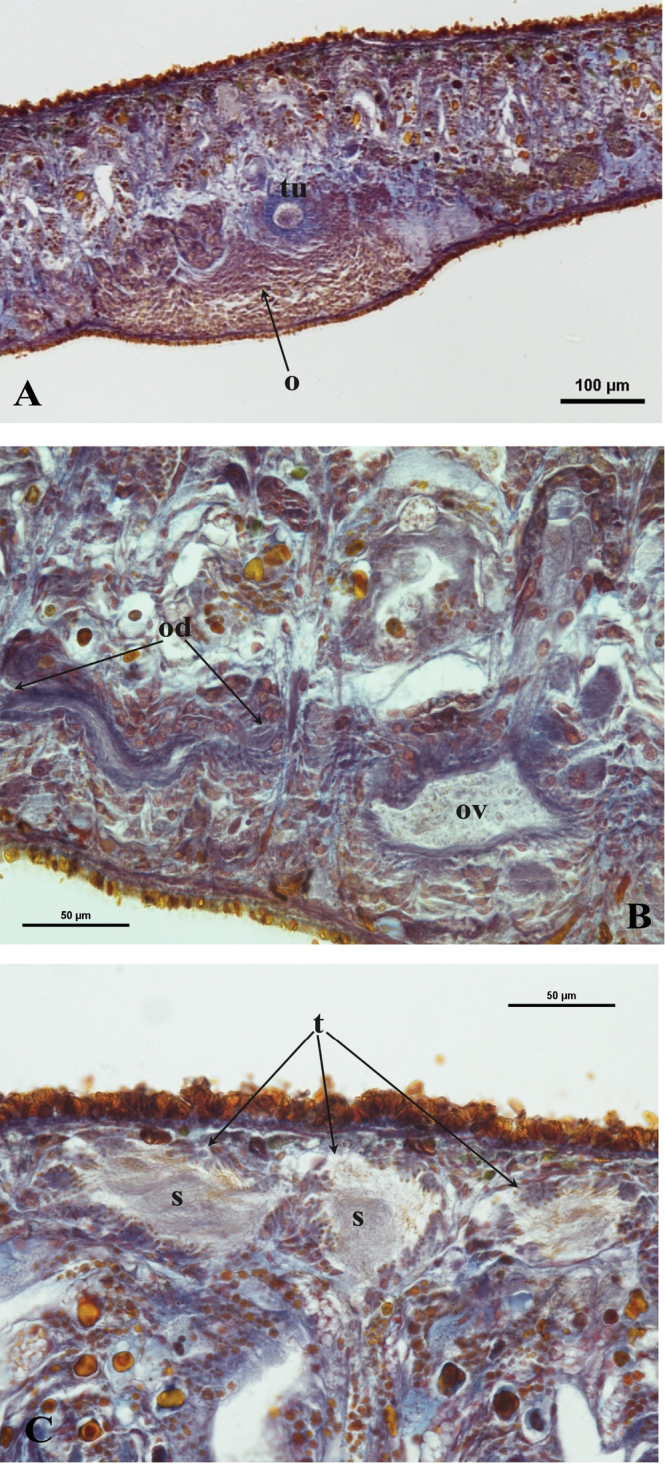
*Dugesia bifida*. **A** Holotype ZMA V.Pl. 7189.1, microphotograph of the right hyperplasic ovary (o) with the tuba (tu) **B** Paratype CGAS Pla 7.1, microphotograph of the oviduct (od) with an expansion (ov) **C** Holotype ZMA V.Pl. 7189.1, microphotograph of mature testes (t) with sperm (s).

The ovaries, localized just behind the brain, are weakly hyperplasic. They occupy half of the dorso-ventral space of the body and are particularly expanded in horizontal direction. The anterior portion of the infranucleated oviducts is expanded into a tuba that may communicate, at a poorly defined position, with the dorsal side of the ovaries or with the center of the ovarian masses, dependent upon the hyperplasic condition of the ovaries (Fig. 6A). The oviducts run ventrally in a caudal direction to beyond the level of the genital pore and, subsequently, recurve anteriad to open at the same level into a long, posterior duct with an histology similar to that of an oviduct. For descriptive purposes we consider this to be a common posterior oviducal extension. The right oviduct opens dorsally into this long common duct while the left oviduct opens ventrally. From this point the common posterior duct divides into two branches, which open separately and asymmetrically through the posterior wall of the bursal canal. The left branch opens slightly dorsally to the right one. The openings of these two branches into the vertically running section of the bursal canal are situated close together (Figs 3A, B, 4). The lumen of the common posterior oviducal extension, and also that of the two branches contains ample sperm. In CGAS Pla 7.1 specimen the most posterior part of the common posterior oviducal extension communicates through a thin ductule with the ventral part of an adjacent vitellarium (Fig. 5C). In the holotype ZMA V.Pl. 7189.1 and in specimens CGAS Pla 7.1 and CGAS Pla 7.2 the lumen of the oviducts has an irregular diameter and is generally quite spacious, thus in some parts forming a kind of vesicle (Fig. 6B).

The numerous mature, fully developed testes are situated dorsally and extend from the level of the ovaries to the posterior end of the body. Spermatogenesis appears to proceed in a regular fashion, in that no anomalies, such as irregularly shaped spermatids and spermatozoa, were observed (Fig. 6C). Vitellaria are located between the testes and the intestinal branches and extend to some distance posteriorly to the copulatory apparatus.

The large copulatory bursa is lined by a columnar, glandular epithelium bearing basal nuclei and it is surrounded by a layer of muscles. In the holotype ZMA V.Pl. 7189.1 a spermatophore full of sperm is present in the lumen of the bursa. From the postero-dorsal wall of the bursa the bursal canal runs in a caudal direction to the left of the copulatory apparatus and, after a narrowing, communicates with the common atrium. The bursal canal is lined with cylindrical, infranucleated, ciliated cells and is surrounded by a thin subepithelial layer of longitudinal muscles, followed by a layer of circular muscles. Ectal reinforcement is absent. The very abundant shell glands open into the vaginal section of the bursal canal, at the level of the oviducal openings (Figs 3A, 5B).

The scarcely developed penis bulb, rich in glands, consists of intermingled longitudinal and circular muscle fibres. Extra-bulbar penial glands (staining yellow with Mallory-Cason) penetrate the penis bulb at its dorsal and ventral side. The penis bulb houses a very large, flask-shaped seminal vesicle, lined with a nucleated epithelium (Figs 3A, 5A). The vasa deferentia penetrate the proximal, anterior section of the penis bulb and open separately and asymmetrically into the seminal vesicle at a position about halfway along the vesicle, at the point where it narrows. The right vas deferens opens dorsally to the left one. The seminal vesicle opens into the ejaculatory duct via a large, valve-like diaphragm. In all specimens examined the sperm ducts form well-developed spermiducal vesicles, packed with sperm. These vesicles are very long and extend over a large distance, viz. from the root of the pharynx to the penis bulb. The diaphragm, located approximately at the base of the penis papilla, receives the openings of penis glands. The stubby, asymmetrical penis papilla is covered by an infranucleated epithelium that is underlain with a subepithelial layer of longitudinal muscles. The ejaculatory duct follows a ventral course and has a terminal opening. A ventrally displaced course of the ejaculatory duct is present in all specimens examined, albeit that this condition is more clearly expressed in some specimens as compared to others, depending on the state of contraction of the penis papilla. For example, in paratype V.Pl. 7189.5 the penis papilla is cone-shaped and shows a distinctly ventrally displaced ejaculatory duct, with a terminal opening. A similar situation is present in paratype CGAS Pla 7.2. In contrast, in the holotype and in paratype CGAS Pla 7.1 the penis papilla is much more stubby, due to contraction, with the result that the ventral course of the ejaculatory duct is much less pronounced. The ejaculatory duct, which in most of the specimens examined contained an empty spermatophore, is lined by a cuboidal, infranucleated epithelium (Figs 3, 5A).

The genital atrium is divided into a common atrium and a male atrium and is lined by an infranucleated epithelium that is underlain by a subepithelial layer of circular muscles, followed by a layer of longitudinal muscle fibres. The common atrium opens ventrally through the gonopore, which receives the openings of the cement glands (Figs 3A, B, 5A, B).

### Karyology

**Figure 7.**
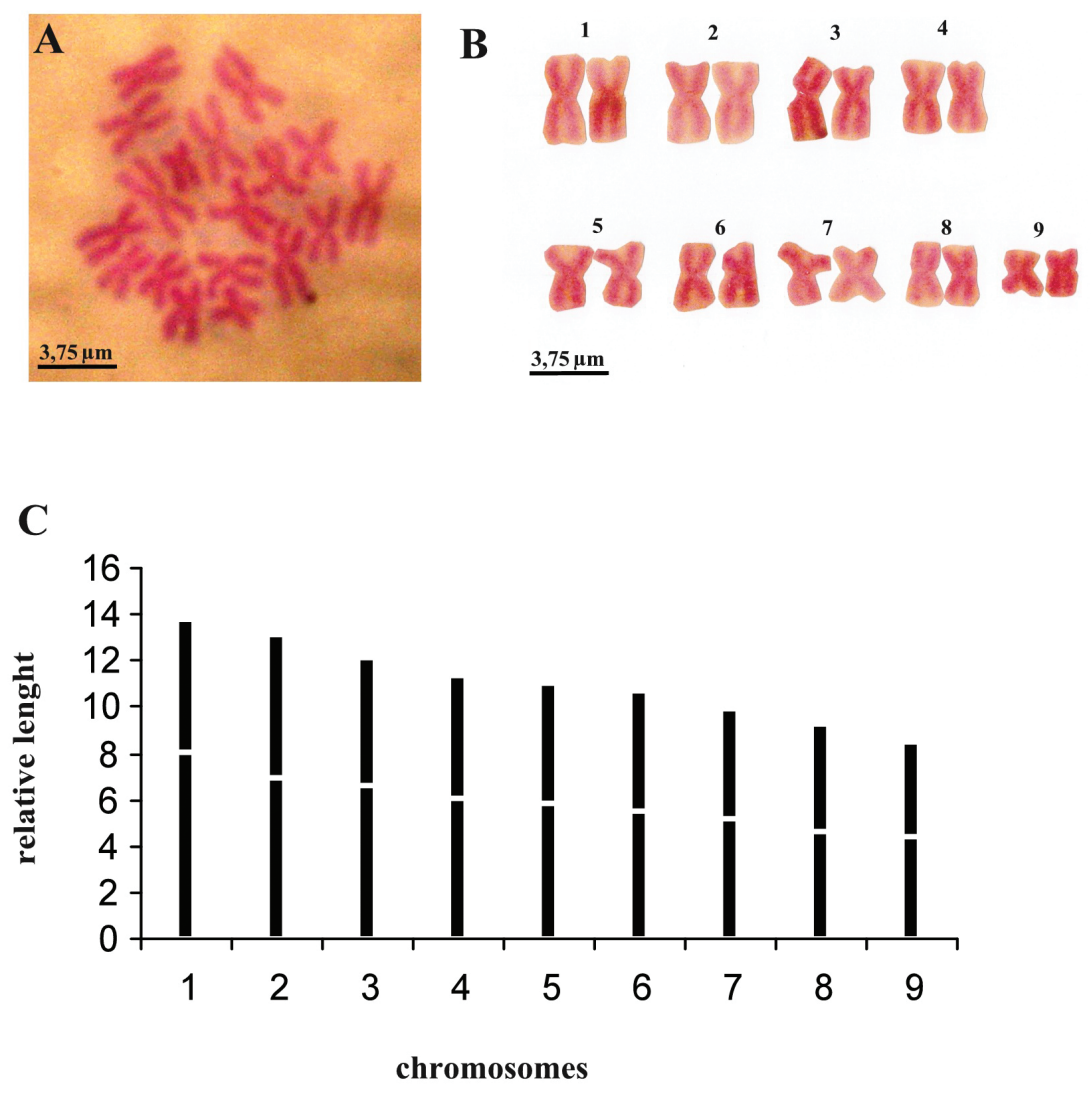
*Dugesia bifida*. **A** metaphasic plate **B** karyogram **C** idiogram.

Metaphasic plates revealed that the specimens constantly showed a set of 18 chromosomes. Chromosomes from six metaphasic plates could be arranged, according to their length, into nine groups of two chromosomes with a diploid chromosome set of 2n = 18; n = 9. Analysis within each group of chromosomes revealed uniformity in both length and centromeric position. Chromosomal length decreases gradually, with low standard deviation values. Centromeric indices showed great variation, in particular for some chromosomes that exhibit high standard deviations, such as numbers 1 and 3 (Fig. 7, Table 2).

**Table 2.** *Dugesia bifida*. Mean values and standard deviations of the relative length (r. l.) and centromeric indices (c. i.).

Chromosome									
	1	2	3	4	5	6	7	8	9
r.l.	13.84 ± 0.68	13.12 ± 0.41	12.14 ± 0.45	11.38 ± 0.16	11.06 ± 0.34	10.65 ± 0.40	9.98 ± 0.37	9.27 ± 0.20	8.49 ± 0.57
c.i.	41.55 ± 3.40	47.04 ± 1.73	45.46 ± 3.35	47.04 ± 1.04	47.54 ± 2.72	48.36 ± 2.68	48.23 ± 1.57	49.50 ± 1.20	48.06 ± 1.59

The karyometric data indicate that the diploid chromosome complement is characterized by metacentric heterobrachial chromosomes, with the exception of chromosome 8, which is metacentric, bordering on metacentric isobrachial (Table 2).

### Life cycle

The life cycle was monitored for two years under laboratory conditions. All 22 individuals were asexual at collection at the end of the dry season of a particularly arid year (September, 2011). In the laboratory the strain notably increased in numbers due to asexual reproduction by fission. After having been kept in the laboratory for about eight months fissiparous specimens displayed a sexualization process (ca. 4%) producing ex-fissiparous individuals characterized by hyperplasic ovaries, large body size and development of the copulatory apparatus. This sexualization process, as well as mating, occurred from spring to summer (May-September), followed by fertile cocoon deposition from June to November. After a growth phase of ca. three months, the juveniles divided repeatedly, thus producing new fissiparous clones. After cocoon deposition, the ex-fissiparous individuals resorbed the copulatory apparatus and returned to the fissiparous mode. All cocoons are characterized by the absence of a pedicel, and were cemented firmly to the substratum, i.e. to the wall and bottom of the bowls in which the animals were reared.

## Discussion

*Dugesia bifida* differs from its congeners in its external morphology, in particular the head shape, the peculiar course of the oviducts at the level of the copulatory apparatus, including the presence of a common posterior extension, and in the asymmetrical openings of the vasa deferentia at about halfway along the seminal vesicle. Further, it is characterized also by a ventral course of the ejaculatory duct with a terminal opening, a large seminal vesicle, and unstalked cocoons.

Almost all known species of *Dugesia* are characterized by a distinctly triangular head with pointed auricles, whereas in *Dugesia bifida* the latter are much more smoothly rounded. The only other species of *Dugesia* with a peculiar external morphology is *Dugesia milloti*, which is characterized by a head with a high triangular shape and prominent, pointed auricles. The latter species is known only from Madagascar and from the island of Anjouan in the Comoros Archipelago (De Beauchamp 1952, De Vries 1988). It is remarkable that these two *Dugesia* species with an aberrant external morphology both occur on Madagascar.

The peculiar condition of the oviducts in *Dugesia bifida* lies in the fact that the ducts open into a common posterior extension, which anteriorly divides into two branches before opening into the vaginal section of the bursal canal. Such a condition was never reported before for the genus *Dugesia*. Usually, in this genus the oviducts run in caudal direction and at the level of the copulatory apparatus open symmetrically or asymmetrically into the vaginal part of the bursal canal. Exceptions to this rule are *Dugesia myopa* and *Dugesia congolensis* De Beauchamp, 1951 from the Afrotropical Region, *Dugesia mertoni* (Steinmann, 1914) from the Australasian region, *Dugesia deharvengi* Kawakatsu & Mitchell, 1989, and *Dugesia andamanensis* (Kaburaki, 1925) from the Oriental Region, in which the oviducts fuse to form a short common oviduct before opening into the bursal canal. Two other species, viz. *Dugesia lindbergi* De Beauchamp, 1959 from the Palaearctic and Oriental regions and *Dugesia uenorum* Kawakatsu & Mitchell, 1995 from the Australasian Region are characterized by a polymorphism concerning symmetrical openings and a common oviducal condition (cf. Sluys et al. 1998).

That in *Dugesia bifida* the common posterior extension belongs to the oviducts is demonstrated by the fact that the histological architecture of this common duct is the same as the rest of the oviducts. The continuation of the oviducts caudally to the copulatory apparatus may be functionally related to the presence of vitellaria in the tail region.

This common posterior extension of the oviducts in *Dugesia bifida* reminds one of the caudally branched oviducts reported for some other genera of the Dugesiidae viz. *Spathula* Nurse, 1950 from New Zealand and Australia, *Reynoldsonia* Ball, 1974, and *Eviella* Ball, 1977 from Australia, and three species of the genus *Romankenkius* Ball, 1974 from Tasmania (cf. De Vries and Sluys 1991, Sluys 2001). According to Sluys' (2001) phylogenetic analysis, presence of caudally branched oviducts could be considered as a synapomorphy for a group comprising the genera *Spathula*, *Eviella*, and *Reynoldsonia*, albeit that there are also three cases of parallelism, viz. *Romankenkius sinuosus* Sluys & Kawakatsu, 2001, *Romankenkius libidinosus* Sluys & Rohde, 1991, and *Romankenkius pedderensis* Ball, 1974 [note that this interpretation differs somewhat from that discussed by Sluys (2001, p. 70)].

Caudally branched oviducts occur also in other species belonging to different freshwater families, such as the dendrocoelid *Macrocotyla glandulosa* Hyman, 1956 and the cavernicolan *Rhodax evelinae*, Marcus, 1946 (cf. Sluys 2001).

Among African and Madagascan species of *Dugesia* absence of ectal reinforcement in *Dugesia bifida* is shared only with *Dugesia aethiopica* Stocchino et al., 2002 and *Dugesia afromontana* Stocchino & Sluys, 2012 (Stocchino et al. 2002, 2012).

From Madagascar only three species of *Dugesia* have been reported up to this moment, viz. *Dugesia debeauchampi*, *Dugesia milloti*, and *Dugesia myopa* (De Beauchamp 1952, De Vries 1988) (Table 1). In contrast to *Dugesia bifida*, a central course of the ejaculatory duct is displayed by these three species, while *Dugesia myopa* also has a short common oviduct and reduced eyes. *Dugesia bifida* shares with *Dugesia milloti* and *Dugesia myopa* a large diaphragm, whereas *Dugesia debeauchampi* has a small diaphragm.

As for life history, the life cycle of *Dugesia bifida* under laboratory conditions is comparable to that of other African species, such as *Dugesia aethiopica* and *Dugesia afromontana*, in which post–pharyngeal transverse fissioning occur continuously, while sexual reproduction followed by fertile cocoon deposition is less frequent, involving only a small percentage of individuals. In contrast to *Dugesia aethiopica*, ex-fissiparous specimens of *Dugesia bifida* do not retain the fissioning ability during their sexual state (cf. Stocchino and Manconi 2013).

In those species of *Dugesia* in which ex-fissiparous specimens develop from fissiparous strains, these sexualized individuals are characterized by the presence of hyperplasic ovaries and underdeveloped testes, in which germ cells show degenerative processes (cf. Stocchino et al. 2012). In contrast, in *Dugesia bifida* the testes are well developed in all specimens examined, without anomalies of the germ cells. Moreover, the hyperplasic ovaries in *Dugesia bifida* are not visible through the dorsal body wall of living animals, in contrast with other species, such as *Dugesia sicula* Lepori, 1948, *Dugesia maghrebiana* Stocchino et al., 2009, *Dugesia aethiopica*, *Dugesia afromontana*, and *Dugesia arabica* Harrath & Sluys, 2013 (Stocchino et al. 2012, Stocchino and Manconi 2013, Harrath et al. 2013). This may be due to the fact that in *Dugesia bifida* the hyperplasic ovaries are more weakly developed and more expanded in horizontal direction than in vertical direction.

The cocoons of *Dugesia bifida* are unstalked but firmly attached by cement to the substratum. The production of unstalked cocoons is an uncommon condition in the genus *Dugesia*, for which they are generally reported as being provided with a pedicel and a terminal plate (Gourbault 1972, Ball and Reynoldson 1981). Among dugesiids, stalked cocoons are known also from *Schmidtea* Ball, 1974 (cf. Gourbault 1972), *Cura* Strand, 1942 (Ball 1974, Grant et al. 2006), and *Girardia dorotocephala* (Woodworth, 1897) (cf. Gourbault 1972). In *Spathula ochyra* Ball & Tran, 1979 the cocoons are enclosed in a jelly-like dome, attached to the substratum (Grant et al. 2006). Unstalked cocoons that can be fastened to the substratum very lightly or attached firmly to it by cement, are reported for the dugesiid genus *Neppia* Ball, 1974, the planariid genera *Phagocata* Leidy, 1847 and *Seidlia* Zabusov, 1911, and the dendrocoelid genus *Dendrocoelopsis* Kenk, 1930. Specifically, unstalked cocoons have been reported for the planariid species *Planaria torva* (Müller, 1774), *Polycelis nigra* (Müller, 1774), *Polycelis tenuis* Ijima, 1884, *Polycelis felina* (Dalyell, 1814), and *Crenobia alpina* (Dana, 1776), and for the dendrocoelids *Bdellocephala punctata* (Pallas, 1774), *Dendrocoelum lacteum* (Müller, 1774), *Dendrocoelum album* (Steinmann, 1910), *Dendrocoelum romanodanubialis* (Codreanu, 1949), and *Dendrocoelum vesiculosus* Stocchino & Sluys, 2013, (Gourbault 1972, Ball 1974, Ball and Reynoldson 1981, Stocchino et al. 2013, Kawakatsu pers. comm., Stocchino pers. obs.).

As for karyology, *Dugesia bifida* shows a diploid chromosome complement of 18 chromosomes with basic number n = 9. Among *Dugesia* species this basic number is shared by only six other species: *Dugesia sicula*, *Dugesia maghrebiana* and *Dugesia biblica* Benazzi & Banchetti, 1972, from the Mediterranean region; *Dugesia arabica* from Yemen; *Dugesia aethiopica* and *Dugesia afromontana* from the Afrotropical region (cf. Stocchino et al. 2004, Stocchino et al. 2012, Harrath et al. 2013).

*Dugesia bifida* represents the easternmost record of a species with a basic chromosomal number n = 9. However, *Dugesia bifida* differs from all of these other species in that it is the only species in which fissiparous specimens exhibit a diploid chromosome complement. *Dugesia sicula* and *Dugesia biblica* have sexual and fissiparous populations with diploid and triploid chromosome complements, respectively (Pala et al. 1995, Stocchino et al. 2012). *Dugesia afromontana* is known only from two fissiparous populations with a triploid chromosome complement (Stocchino et al. 2012). *Dugesia maghrebiana* is represented by a fissiparous population, characterized by a condition of diffuse mosaicism or mixoploidy, with each individual having triploid and tetraploid cells (Stocchino et al. 2009). The only fissiparous population of *Dugesia aethiopica* shares with *Dugesia maghrebiana* the condition of mixoploidy, but chromosome complements of the former species are diploid and triploid. *Dugesia arabica* shows a combination of the above-mentioned conditions, in that it has diploid populations that reproduce sexually, triploid fissiparous populations, and mixoploid (diploid and triploid) populations reproducing sexually as well as by fission (Harrath et al. 2013).

With respect to this group of six species discussed above, it turns out that fissiparous populations are always triploid or mixoploid, while sexual populations are diploid.

The presence of well developed testes with very abundant sperm and weakly hyperplasic ovaries in ex-fissiparous specimens of *Dugesia bifida* may be related to its diploid condition, in that it allows a more regular meiosis. A similar condition was reported for *Dugesia colapha* Dahm, 1967 from Ghana, in which the fissiparous populations that produced ex-fissiparous individuals under laboratory conditions were characterized by regular diploid chromosome complements (2n = 16; n = 8). However, in this case, ex-fissiparous individuals were always sterile, despite their regular gametogenesis (Dahm 1967).

## Supplementary Material

XML Treatment for Dugesia bifida
